# Autonomous visual exploration creates developmental change in familiarity and novelty seeking behaviors

**DOI:** 10.3389/fpsyg.2013.00648

**Published:** 2013-09-20

**Authors:** Sammy Perone, John P. Spencer

**Affiliations:** Department of Psychology and Delta Center, University of IowaIowa City, IA, USA

**Keywords:** visual exploration, dynamic systems, dynamic neural fields, intrinsic motivation

## Abstract

What motivates children to radically transform themselves during early development? We addressed this question in the domain of infant visual exploration. Over the first year, infants' exploration shifts from familiarity to novelty seeking. This shift is delayed in preterm relative to term infants and is stable within individuals over the course of the first year. Laboratory tasks have shed light on the nature of this familiarity-to-novelty shift, but it is not clear what motivates the infant to change her exploratory style. We probed this by letting a Dynamic Neural Field (DNF) model of visual exploration develop itself via accumulating experience in a virtual world. We then situated it in a canonical laboratory task. Much like infants, the model exhibited a familiarity-to-novelty shift. When we manipulated the initial conditions of the model, the model's performance was developmentally delayed much like preterm infants. This delay was overcome by enhancing the model's experience during development. We also found that the model's performance was stable at the level of the individual. Our simulations indicate that novelty seeking emerges with no explicit motivational source via the accumulation of visual experience within a complex, dynamical exploratory system.

One of the oldest questions in the history of human thought is what motivates an individual to achieve a level just beyond reach. Such motivation appears to be a central quality of human behavior, and may be a driving force behind scientific advancement, corporate innovation, and more generally, cultural evolution. Striving beyond one's reach is also an apt characterization of human development where children undergo a series of astonishing transformations. The newborn has a limited repertoire including sleeping, eating, and crying. By the end of the first year, the infant can walk and is beginning to talk. By age 5, the child is learning to read, write, and sit in a classroom among peers. What motivates a child to accomplish so much in so little time?

Seminal theories of cognitive development posit that infants' active exploration of their environments enables them to develop skilled action and cognitive systems (Piaget, 1952; Gibson, 1988). Infants are seemingly driven to act by curiosity, ambiguity, and novelty. These forces characterize intrinsic motivation and are widely held in developmental psychology to propel development forward (for a review, see Oudeyer and Kaplan, 2007). Yet the nature of intrinsic motivation and the mechanisms by which it creates change remain unclear.

Infancy might offer unique insights into the very nature of intrinsic motivation and its role in development. But how do we investigate intrinsic motivation in infants who have a limited behavioral repertoire? This requires clever methods to assess how infants think. Such methods first emerged in the 1970s when researchers developed a battery of novel habituation paradigms that relied on infants' looking behavior to measure cognition (Cohen, 1972a,b; Fantz, 1974). In these paradigms, infants are given experience looking at one item in isolation or in pairs. Then, infants' preference to look at a novel item relative to the familiar item is measured. Infants' preference to look at a familiar over a novel item is taken as evidence that they recognize the familiar item but have not yet formed a robust memory for it. Infants' preference for novelty is taken as evidence that they have formed a robust memory for the familiar item and are beginning to learn about the novel item.

The use of looking paradigms led to the accumulation of a vast literature on infant cognition. A key finding from this literature is that infants' familiarity and novelty preferences change across multiple timescales, including during learning within a task and over weeks, months, and years in development (for reviews, see Hunter and Ames, 1988; Rose et al., 2004). With only brief exposure to a stimulus, infants will exhibit a familiarity preference. After prolonged exposure to the stimulus, infants will exhibit a novelty preference (Rose et al., 1982; for exceptions and detailed analysis, see Roder et al., 2000; Fisher-Thompson and Peterson, 2004). Critically, the rate at which infants move through this familiarity-to-novelty shift increases with age. In fact, during the first 1–2 months of life, infants move through this shift so slowly that they sometimes show no novelty preference even after several minutes of exposure (Wetherford and Cohen, 1973; Fantz, 1974). With age, however, infants spend more time looking at novel items relative to familiar items (Fantz, 1974).

This characterization of familiarity and novelty preferences is, of course, somewhat oversimplified. Infants' preferences are influenced by stimulus conditions, for instance (for reviews, see Hunter and Ames, 1988; Rose et al., 2004). For some stimuli, infants show no evidence of familiarity preferences early in learning (Roder et al., 2000). For other stimuli, infants show a familiarity preference late in learning (Shinskey and Munakata, 2005). To complicate matters further, some studies have shown that individual infants oscillate between familiarity and novelty as they explore items (Fisher-Thompson and Peterson, 2004). And even adults will show familiarity preferences under conditions in which they freely explore visual scenes (Dodd et al., 2009). Thus, the same exploratory system appears to organize itself differently across contexts.

In the present report, we focus on the robust, quantitative increase in infants' exploration of novelty over development (for a broader theoretical evaluation of the familiarity-to-novelty shift, see Perone and Spencer, 2013a). This shift has been attributed to an increase in visual processing speed over development. Rose et al. (2002) nicely quantified this shift using a processing speed task with 5-, 7-, and 12-month-old infants. Infants were presented with pairs of different stimuli across trials. On each trial, one stimulus remained unchanged (familiar) and one changed (novel). This design enabled Rose et al. to quantify the time infants' spent looking at the familiar item before shifting over to explore novel items. Processing speed was measured as the number of trials to a criterion defined as a looking preference for the novel item on three consecutive trials. With age, infants accumulated less time looking to the familiar item and more quickly shifted toward looking to the novel item. This resulted in a reduction in the number of trials to reach criterion over development.

The use of looking paradigms has also led to two other key observations. First, infants' birth status influences the development of the familiarity-to-novelty shift. For example, Rose et al. (2002) found that term and preterm infants exhibited different patterns of familiarity and novelty seeking over development. At each age group, preterm infants required more trials to criterion than term infants. Thus, preterm infants exhibited stronger familiarity seeking biases than term infants and those persisted over development. Second, individual differences in looking behavior during infancy are stable over time. For example, Rose et al. (2001; see also Colombo et al., 1987) found that looking measures of exploration (e.g., frequency of gaze switching) and recognition (e.g., preference for novelty) are stable within individuals over the course of the first year. In addition, these looking measures during infancy are predictive of cognition during toddlerhood (Rose et al., 2009) and children's executive functioning at age 11 (Rose et al., 2012).

These laboratory-based observations have shed important light on the nature of the transition from familiarity- to novelty-seeking in the first year. Novelty-seeking has some distinct advantages over familiarity-seeking, enabling infants to explore and acquire knowledge about new items. Moreover, this exploratory process builds a strong base of what is familiar to the infant. But it is not clear from these data what motivates infants to switch their exploratory style. Conceptual and formal theories of infant looking and memory formation have attributed this shift to increases in processing speed (for reviews, see Hunter and Ames, 1988; Rose et al., 2004). By this view, infants' switch in exploratory style is simply a by-product of more efficient processing of visual information in the neural systems involved in doing so (Colombo, 1995). Although compelling, such accounts rarely explain where changes in processing speed come from.

Insights into this question might be obtained by moving from constrained laboratory tasks to less constrained tasks where infants can freely and autonomously explore the world around them. A nice example of this comes from recent studies of the transition from crawling to walking. What motivates an infant to move from skilled crawling to unskilled walking? Why move from an energy-efficient strategy to an energy-inefficient strategy? Adolph et al. (2012; see also Adolph and Robinson, 2013) observed infants' who were learning to walk in more naturalistic settings and made two surprising observations. First, infants engage in massive practice from the onset of walking, walking up to 8 football fields per day. Second, walking is initially as efficient as crawling. Although newly walking infants often fall, they also travel more distance. This observation changes the framing of questions about motivation: if walking is as efficient as crawling, why not walk? Walking creates no additional cost and has many other advantages, enabling infants to carry objects from one location to another and providing a continuous view of the world as they move.

The lesson we take from this work on locomotor development is that questions about transitions in development must be framed within the context of the full range of infants' experiences. Thus, if we want to understand what motivates the infant to move from familiarity- to novelty-seeking over development, we must connect exploration in the laboratory to exploration in the real world. One approach to connecting up these worlds is to evaluate infants' familiarity with items outside of the lab and assess how they learn about those same classes of items inside the lab. For example, Quinn et al. (2002) found that infants' raised by female caregivers were capable of remembering individual female faces in the lab. Similarly, Kovack-Lesh et al. (2008) found that infants raised with pets in the home were capable of remembering individual cat exemplars in the lab. These findings show empirically that the massive visual experience infants acquire outside of the lab is, in fact, a key driver of development. But these are examples of how infants' experience with specific classes of items outside of the lab influences how they form memories for those same classes of items in the lab. Do massive quantities of visual experience in the real world also impact the more general ability to seek novelty?

We examine this possibility in the present report using a novel approach to understanding visual cognition in infancy—computational modeling. Our starting point is an autonomous Dynamic Neural Field (DNF) model of infant looking and learning developed by Perone and colleagues (Perone et al., 2011; Perone and Spencer, 2013a,b). We have used this model in the past to capture data from studies on the familiarity-to-novelty shift. To do this, we changed parameters of the model over development “by hand” to gain an understanding of how this transition might emerge over development. The insight from this work was that general parameter changes in the strength with which excitatory and inhibitory neurons interact in the model transformed an initially familiarity-seeking model into a novelty-seeking model. The key mechanism underlying this change was the emergence of a new ability—the ability to form a working memory (WM) for objects. The ability of the model to quickly form working memories for objects enabled it to recognize those objects as known and explore new objects.

Here, we ask if this model can develop itself and show the autonomous emergence of novelty-seeking behavior. In particular, can we initialize a model with a given set of parameters, situate this model in a virtual world, and let it create its own developmental shift from familiarity- to novelty-seeking via autonomous visual exploration. If so, we can then take a step back and ask: what motivated the model to seek novelty?

In the sections that follow, we describe the DNF model and the hypothesis that guided our “by hand” exploration of development in previous work. We then pursue a demonstration proof that the model can develop autonomously through a variant of Hebbian learning. We do this first at a group level. We created a term infant model, let it develop “outside” the lab, and repeatedly brought the model “into the lab” to assess whether it exhibited the familiarity-to-novelty shift in the processing speed task developed by Rose et al. (2002). Results show that the model effectively captures many aspects of the developmental shift. We also asked whether changes in the initial conditions of the model could mimic the development of preterm infants. Results show that the model captures the developmental delays this infant population exhibits.

These simulations provide an initial demonstration that novelty-seeking can emerge from the accumulation of massive out-of-lab experience in our computational model. But intrinsic motivation is not a group-level phenomenon. The motivation to push boundaries in development happens at the level of the individual infant. Thus, in a second study, we looked at the characteristics of individual simulations and ask whether each simulation creates its own unique path from familiarity- to novelty-seeking. These simulation data provide new insights into the sources of individual differences. We conclude by returning to the issue of intrinsic motivation and raise the possibility that no explicit motivational force is needed to explain developmental change within an autonomously behaving complex neural system.

## A dynamic neural field model of infant visual exploration

**Figure 1:**
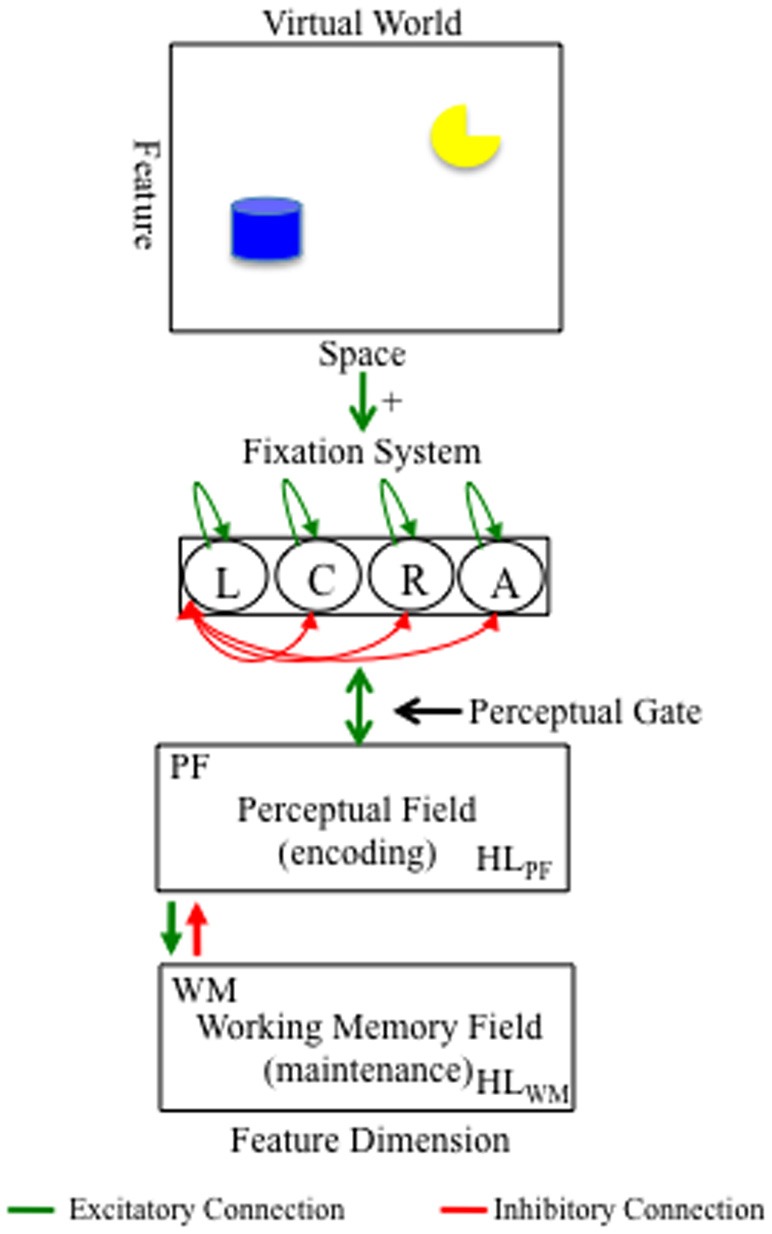
**DNF model architecture.** At the top is a virtual world at which the model looks. The virtual world consists of two objects at left and right locations distributed over a continuous feature dimension (e.g., color). The presence of items at left and right locations bias the fixation system to look at those locations (see green arrow from space to fixation system). The fixation system interacts in a winner-take-all fashion such that fixating a location suppresses fixation to all other locations (see red arrows between nodes). Fixating a location acts like a perceptual gate into the cognitive system, which consists of a perceptual field (PF) and working memory (WM) field. PF and WM are reciprocally coupled to a shared layer of inhibitory interneurons (Inhib; not show). Activity in PF supports fixation (green bi-directional arrow between PF and fixation). Activity in WM suppresses PF via a strongly tuned connection from WM to Inhib (red arrow from WM to PF). Activity in PF and WM are influenced by activity in Hebbian layers, HL_PF_ and HL_WM_, respectively, which accumulates over learning and facilitates encoding in PF and memory formation in WM.

Figure 1 shows the DNF model architecture. Model equations and parameter values are given in the Appendix. For illustration, the model is situated in a virtual world that consists of a typical laboratory setting in which relevant stimuli appear at left and right locations, task-irrelevant stimuli appear at away locations, and attention-getting stimuli often used to orient infants to the location at which stimuli appear at a center location. The fixation system consists of a collection of nodes that fixate left (L), right (R), center (C), and away (A) locations in a winner-take-all fashion. When a node is suprathreshold (>0), it is said to be in the fixation state. The presence of objects in space bias the fixation system to enter the fixation state (see green arrow from space to fixation system).

The fixation system is reciprocally coupled to a neurocognitive system shown in the bottom panels of Figure 1. One component of the neurocognitive system is a perceptual field (PF) that consists of a population of neurons with receptive fields tuned to a continuous feature dimension (e.g., color). The model can represent stimuli along multiple dimensions (see Perone and Spencer, 2013b). For simplicity, we use one dimension here. When a given node in the fixation system is in the fixation state, the stimulus at the associated location is input into PF which encodes the stimulus by forming an activation peak that estimates the feature value (e.g., blue). Neuronal activity within PF is governed by local excitatory/lateral inhibitory interactions. These interactions within PF are relatively weak; thus, once a stimulus is removed, the activation peak relaxes back to the neuronal resting level.

Encoding within PF has two important functions in the model. First, encoding supports continued fixation. Activation in PF feeds back into the fixation system which sustains the fixation state and supports further encoding of the stimulus. Second, encoding leads to the formation of working memories. In particular, activation in PF passes excitatory input to a layer of similarly tuned neurons in a WM field. Like PF, neuronal activity within WM is governed by local excitatory/lateral inhibitory interactions. Unlike PF, however, neural interactions within WM are stronger. Consequently, activation peaks can be maintained in the absence of input via recurrent excitatory and inhibitory interactions. This is the mechanism for maintaining information in WM in the model.

There are two other patterns of connectivity in the DNF model. First, PF and WM are reciprocally coupled to a shared layer of inhibitory interneurons (Inhib; not shown). This connectivity creates the lateral inhibitory interactions within PF and WM. Critically, the connection from WM to Inhib is set such that strong activity in WM suppresses activity in similarly tuned neurons in PF (see red arrow from WM to PF). This weakens support for fixation from PF, leading to the release from the fixation state when a WM peak is present. Thus, the model encodes a stimulus which drives sustained looking and forms a WM for the stimulus which drives looking away. Second, PF and WM are reciprocally coupled to Hebbian layers (HL; not shown) that implement a form of Hebbian learning. In particular, suprathreshold activity in PF and WM leads to the accumulation of activation at similarly tuned sites in HL_PF_ and HL_WM_, respectively. The absence of suprathreshold activity in PF and WM leads to slow decay in these HL. Activation traces in HL_PF_ facilitate encoding of previously encoded stimuli in PF. This supports familiarity-seeking and is the basis of recognizing what is known early in development (Wetherford and Cohen, 1973; Fantz, 1974; Perone and Spencer, 2013a). Activation traces in HL_WM_ facilitate the formation of WM peaks. This can lead to the fast suppression of peaks in PF, freeing the model to look away from familiar or known items toward novel items. Thus, this supports novelty seeking.

**Figure 2:**
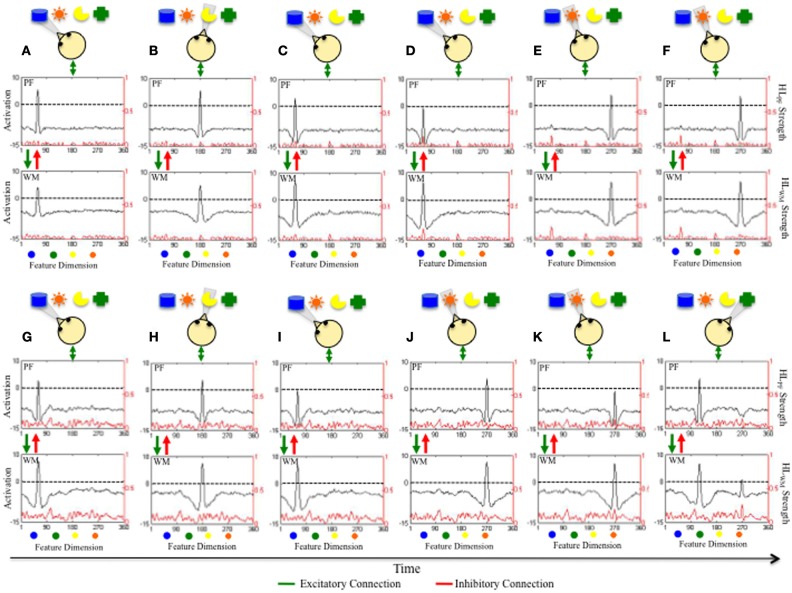
**Illustrates how the accumulation of experience in HL_PF_ and HL_WM_ over a continuous dimension influences real-time exploration of items distributed along that dimension.** Shown at the top of each panel are 5 objects distributed along the color dimension that the model is exploring over time. The cartoon infant shows the direction of gaze. Panels **(A–F)** show how the model explores items after little experience has accumulated in HL_PF_ and HL_WM_ (see red line, right y-axis). Initially, the model is fixating, encoding, and forming a working memory for the blue item (see black line in **A**). The model spontaneously switches gaze and begins to encode and form a working memory for the yellow item **(B)**. The model then looks back to the blue item **(C)**. Dwelling on the blue item leads WM activity to grow in strength and suppress activity in PF **(D)**. This frees the model to switch gaze and encode and form a working memory for a new item, which happens to be the orange item **(E,F)**. Notice the robust long-term memory for the blue item accumulated in HL_PF_ and HL_WM_. Panels **(G–L)** show how the same model explores items after more experience has accumulated in HL_PF_ and HL_WM_ (see red line, right y-axis). Initially, the model is fixating, encoding, and forming and working memory for the blue item **(A)**. The model switches gaze to the yellow item **(B)** before switching back to the blue item **(C)**. Now, the strong experience in HL_PF_ and HL_WM_ enables the model to quickly form a robust memory for the blue item after dwelling for just a short while. The model is freed to explore new items and, again, quickly forms a working memory for the orange item **(J,K)**. The faster rate at which the model encodes and forms memories enables it to explore more items **(L)**.

Figure 2 illustrates the real-time process by which the DNF model learns as it explores objects in a virtual world over time. The top panels show a model that has accumulated little developmental experience exploring items distributed over a color dimension (**A–F**). The bottom panels show the same model after it has acquired more experience (**G–L**). Each panel has the same format. At the top is a collection of objects that the model is exploring over time. The cartoon infant head shows what object is being fixated during each time slice. The next two figures show activation in PF and WM (see black lines and left y-axis) and the strength of experience accumulated in HL_PF_ and HL_WM_ (see red lines and right y-axis).

In Figure 2A, the model first looks at the blue object. This excites neurons in PF which, in turn, supports continued fixation and leads to excitation of similarly tuned neurons in WM. The fixation system is stochastic which enables it to spontaneously disengage fixation and shift gaze direction. In **2B**, the fixation system has switched gaze and is now looking at, encoding, and forming a WM for the yellow object. Notice that activity associated with the blue object has subsided within PF and WM; the model is not encoding the blue object or maintaining a WM of the object. In **2C**, the model has again switched gaze and is fixating the blue object and maintains fixation across **2C,D**. This continued fixation enables the model to form a robust peak in WM and acquire a long-term memory via the HL (see red “bump” of activity in HL_PF_ and HL_WM_ in **2D**). WM activity is also beginning to suppress PF activity to below threshold levels which leads to less support for fixation. Consequently, the model switches gaze. In **2E,F**, the model switches gaze and is fixating, encoding, and forming a WM for the orange object. Once again, the WM of the blue object is not maintained.

In Figures 2G–L, the same model has acquired more experience by exploring a virtual world consisting of objects distributed over a color dimension. This experience has created the stronger, densely distributed traces in HL_PF_ and HL_WM_ shown in **2G–L**. This model is now more familiar with the color dimension. This familiarity has a dramatic impact on looking and learning. In **2G**, the model quickly encodes the blue object into WM, suppressing PF activity to near threshold levels, and biasing the model to switch gaze. In **2H**, the model is fixating the yellow object and, again, WM activity suppresses PF activity to near threshold levels. When the model re-fixates the blue object, WM activity suppresses PF activity to below threshold levels (**2I**) and the model quickly looks away—the model is seeking novelty.

Critically, this novelty seeking behavior is a result of the accumulated long-term experience—the model quickly forms robust working memories because the Hebbian traces have moved WM closer to threshold. This can be seen in **2J–L**. The model fixates the orange object (**J**) and forms a robust memory after maintaining fixation (**K**). This enables the model to explore a new location at which the green object is present (**L**). Notice that WM activity associated with the orange object is hovering around threshold in **2L** even though the model is fixating the green object. This ability to form an enduring, actively maintained WM enables the model to seek novelty, actively contrasting what is known with what is novel. This emerges from a confluence of factors including the duration with which the model fixates an object, the strength of HL_WM_ that facilitates activity within WM, and the strong tuning of local excitatory/lateral inhibitory interactions within WM. This stable WM peak has a dramatic impact of the model's behavior. For example, when the model re-fixates items that it is actively maintaining in a WM state, PF activity is quickly suppressed. This leads to the quick release of fixation and frees the fixation system to seek novel items.

## Simulation experiment 1

The goal of Simulation Experiment 1 was to probe whether the model could develop novelty-seeking behavior from autonomous visual exploration in a “real” world. If so, this might shed light on where the motivation to seek novelty comes from. As described previously, this goal emerged from our earlier work using the DNF model to quantitatively simulate the familiarity-to-novelty shift in early development (Perone and Spencer, 2013a). We did this by changing parameters of the model “by hand” according to the spatial precision hypothesis (SPH) proposed by Schutte and Spencer (2009; see also Schutte et al., 2003; Simmering et al., 2007; Perone et al., 2011; Perone and Spencer, 2013a,b).

According to the SPH, excitatory and inhibitory interactions become stronger over development, leading to more robust neural activation states and “sharper” peaks of activation. Implementing the SPH involves strengthening within-layer excitatory connections in PF and WM and cross-layer inhibitory interactions from Inhib to PF and WM. When neural interactions are weak, the model slowly encodes and slowly forms peaks in WM. This creates a familiarity-seeking model that dwells on familiar items for relatively long durations before looking to novel items. When neural interactions are stronger, the model quickly encodes items and quickly forms peaks in WM. This creates a novelty-seeking model that has short dwell times on familiar items before looking to novel items (see Perone and Spencer, 2013a).

Implementing the SPH in the DNF model only requires changes in the strength of excitation and inhibition. Might these changes emerge from a simple Hebbian learning process? Recall that HL coupled to PF and WM accumulate memory traces as peaks are built in PF and WM. This increases the excitability of previously active sites as well as nearby sites based on a similarity gradient. As general experience across a dimension accumulates, this might approximate the increase in excitatory strengths we implemented by hand. What about the increase in inhibition? As excitatory interactions strengthen, PF and WM will pass stronger activation to the shared inhibitory layer. This might give rise to an effective increase in inhibition as well.

We explore these possibilities here across two groups of simulations. In one set of simulations, the DNF model was tuned to mimic the behavior of term infants. In the second set of simulations, the model was tuned to be “less mature” using the SPH as a guide. This enabled us to examine how the initial conditions set by the model parameters impact development relative to the role of massive “out-of-lab” experience. To benchmark these simulations, we assessed the familiarity- and novelty-seeking biases of the model in the processing speed task developed by Rose et al. (2002) by repeatedly bringing the model “into the lab” over the course of its development.

**Figure 3:**
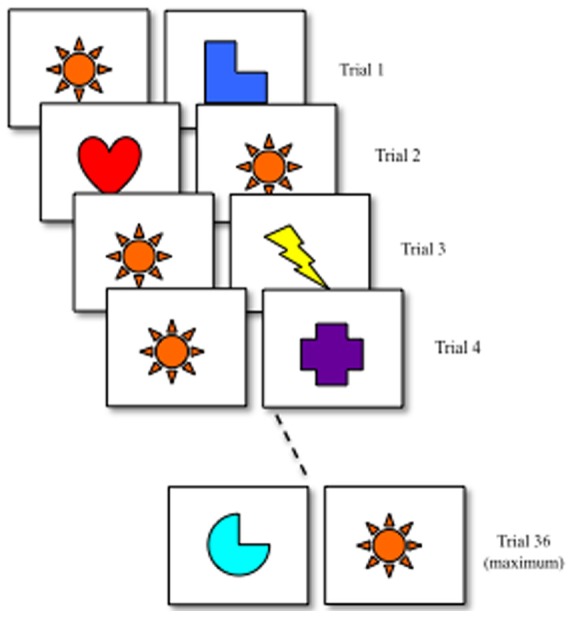
**Processing speed task developed by Rose et al. (2002).** Infants were presented with a pair of different stimuli on each trial. Across trials, one stimulus remained unchanged (familiar) and one changed (novel). On each trial, infants were required to accumulate 4 s of looking. Infants met a learning criterion once they looked at the novel stimulus more than 55% of the time on the 3 consecutive trials or 36 trials had passed. In the empirical study, stimuli were faces. There were 19 stimuli, one designated as the familiar and 18 designated as novel. If 18 trials had passed before infants met the criteria, the 18 novel stimuli were represented.

Figure 3 shows a schematic of the processing speed task. At the beginning of the task, infants are presented with a pair of different stimuli. In Rose et al. (2002), faces were used as stimuli. The procedure has been used in other studies as well and is robust to variation in stimuli (Robinson and Sloutsky, 2007). After the first trial, one item was designated as the familiar item and remained unchanged across trials (orange star). Infants were required to accumulate 4 s of looking on each trial. Once infants met the looking criterion, the trial ended and the next trial began. On each trial, a novelty score was calculated by dividing looking to the novel stimulus by total looking accumulated across the novel and familiar stimulus. The measure of processing speed was the number of trials required to exhibit a novelty score greater than 55% on three consecutive trials.

Rose et al. (2002) reported three additional measures of looking. The first is looking to the familiar item which is the amount of time infants accumulated looking to the familiar stimulus prior to meeting criteria. This is a good index of infants' familiarity seeking bias and has long been assumed to reflect the time required for infants to form memories (Cohen, 1972a,b; Hunter and Ames, 1988; Colombo and Mitchell, 1990). The second is shift rate which is the rate of gaze switching relative to time spent looking. Shift rate has been proposed to reflect the efficiency with which infants distribute their attention through time and space (Rose et al., 2007). The last is look duration which the average length of each look. Like shift rate, look duration has been proposed to be a measure of disengaging and distributing attention (Rose et al., 2007).

**Figure 4:**
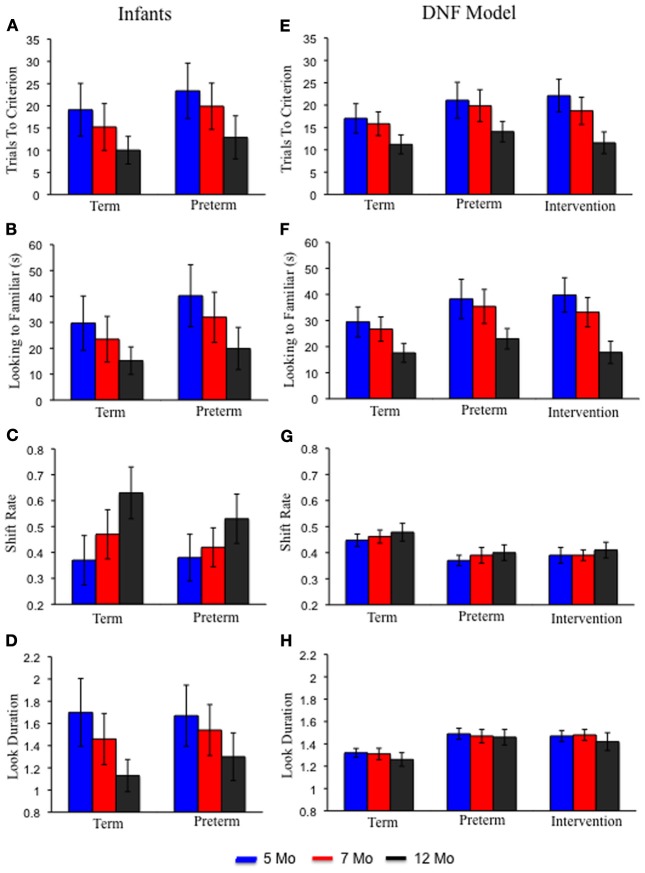
**Panels (A–D) show empirical results from the processing speed task reported by Rose et al. (2002) for term (left) and preterm (right) infants at 5 (blue), 7 (red), and 12 (black) months of age.** With age, term and preterm infants exhibited fewer trials to criterion **(A)**, accumulated less time looking to the familiar **(B)**, higher shift rates **(C)**, and shorter look durations **(D)**. Preterm infants' behavior at every age resembled that of younger, term infants. Panels **(E–H)** show results from the DNF model in the processing speed task for the term (left), preterm (middle), and intervention (right) models. The DNF model exhibited a similar pattern of results for the term and preterm infant models. The intervention model showed performance that resembled the term model by 12 months of age.

Figures 4A–D shows infants' performance in the processing speed task (Rose et al., 2002). The left portion of each panel shows term infants at 5 months of age (blue bars), 7 months of age (red bars), and 12 months of age (black bars). Over development, term infants exhibited a decrease in trials to reach criterion **(A)**, accumulated less time looking to the familiar item **(B)**, exhibited higher shift rates **(C)**, and exhibited shorter look durations **(D)**. Preterm infants produced a similar pattern of results but, critically, at each age exhibited behavior that resembled relatively younger term infants. For example, 7-month-old preterm infants required about the same number of trials to reach criterion as 5-month-old term infants. This pattern of results indicates that preterm infants are delayed on these measures.

In the past, we have used the DNF model and SPH to capture developmental changes in the suite of measures assessed by Rose et al. (2002) using data from a preferential looking paradigm (Perone and Spencer, 2013a). Here, we test whether the accumulation of experience in the DNF model can do the work of the SPH and quantitatively simulate the empirical data shown in Figure 4.

### Method

The DNF model was situated in a simple virtual world consisting of two items that varied along a single dimension. The dimension consisted of 360 degrees of metrically organized continuous feature space (e.g., color). We randomly sampled items for the model to explore from a set of 360. A non-fixated item was replaced every 1000 time steps. This enabled the model to sample many different items over time, consistent with what infants might experience interacting with parents as they show infants different toys from a larger set of possible toys.

The simulations were parsed into 30 10,000 time step episodes of visual exploration (300,000 time steps of experience in total). Conceptually, these episodes occur over the time scale of months; however, in the model, we condensed this experience considerably to keep the simulation time reasonable (e.g., even with this condensed “out-of-lab” experience, it took over 8 h of simulation time to run a single simulation through the full set of out-of-lab and in-the-lab experiences). In addition to the 30 episodes of exploration, we inserted inter-episode intervals of 100 time steps. During these intervals PF, WM, and Inhib were re-initialized (i.e., set to 0 activation). This eliminated any sustained WM peaks and reset the fields for exploration of new items at the onset of the next episode.

We wanted to test whether differences in the initial conditions of the DNF model could account for population differences in the familiarity-to-novelty shift over development. Thus, we created two models with differences in the initial parameter values using the SPH as our guide. Specifically, we first created a “term” model. To do this, we allowed the DNF model to accumulate experience in the HL by exploring a virtual world and assessed its performance in the processing speed task over the course of its development (see below). We then hand-tuned the DNF model parameters until we established a parameter set that produced a pattern of results that was quantitatively similar to the empirical results for the term infants reported by Rose et al. (2002). After that, we uniformly weakened the SPH parameters by 20%. We will refer to this weaker parameter set as the “preterm” model.

**Figure 5:**
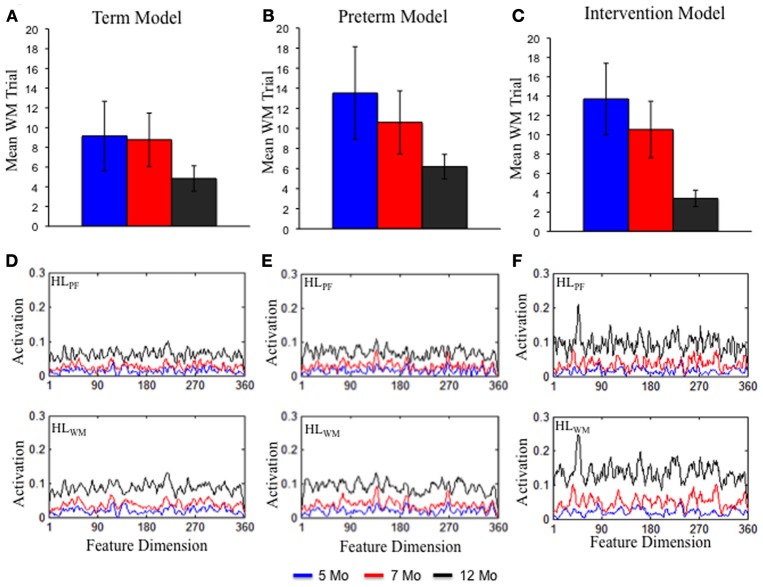
**The top shows the rate at which DNF model formed a stable WM peak for the term (A), preterm (B), and intervention (C) models at 5 (blue), 7 (red), and 12 (black) months of age.** Over development, all models formed a stable WM peak more quickly. The rate of WM peak formation was delayed for the preterm model but enhanced by 12 months of age for the intervention model. The bottom shows the experience accumulated in HL_PF_ and HL_WM_ for the term **(D)**, preterm **(E)**, and intervention **(F)** models. The strength of activation in the Hebbian layers was comparable for the term and preterm infant models. It was stronger for the intervention model.

The development of the term and preterm models were simulated 5 times each. During each simulation, we saved the state of HL_PF_ and HL_WM_ after each episode of exploration. We then averaged HL_PF_ and HL_WM_ across all 5 simulations. This created nearly uniform levels of activation across all neuronal sites in the HL by smoothing out the peaks and valleys of activation in the layers that were unique to each individual simulation (e.g., compare the HL for group level simulations in Figure 5 to individual simulations in Figure 7). This uniformity mimics the strengthening of excitatory connections across an entire dimension we implemented by hand when we implemented the SPH in previous work. Our goal in averaging the HL was to maximize the stability of the model's behavior across simulations when situated in the processing speed task (see below), much like averaging the looking behavior across a group of infants.

Next, we initialized the term and preterm models with their respective mean HL_PF_ and HL_WM_ accumulated at 5, 10, and 30 episodes and situated each model in the processing speed task developed by Rose et al. (2002). For ease of comparison to the empirical data, we refer to these initializations as the term and preterm infant models at 5, 7, and 12 months of age. We ran 100 simulations of each model. This number of simulations provided a thorough assessment of the range of the model's looking behavior in the processing speed task in the context of the natural variation the model shows when placed in a laboratory-based learning task (for a discussion, see Perone and Spencer, 2013a,b). To precisely map the models' performance in the lab with the timing of events in the speed of processing task, we assumed the mapping used in our previous studies where 200 time steps in the model was equal to 1 s (Perone and Spencer, 2013b). Note that in the simulation method described above, learning inside the lab did not influence the model's performance outside of the lab.

### Results and discussion

The simulation results are presented in the following three sections. In the first section, we describe the DNF model's performance in the processing speed task and the underlying neurocognitive dynamics. In the second section, we probe whether the development of the preterm infant model might be modified through an intervention. This helped us assess the influence of the initial model parameters relative to the accumulation of out-of-the-lab experiences. In the third section, we probe whether the accumulation of experience in the HL led to sharper and more robust WM peaks consistent with the SPH we implemented “by hand” in previous work.

#### Cognitive and behavioral dynamics

Figures 4E–H shows the DNF model's performance in the processing speed task. Like infants, over development the term infant model exhibited a decline in trials to reach criterion (**E**) and accumulated less time looking to the familiar item (**F**). The model also showed a small, quantitative increase in shift rate (**G**) and decrease in look duration (**H**) over development. Like infants, the preterm infant model exhibited a similar, but delayed, pattern relative to the term infant model.

What are the sources of these developmental changes in the model's performance? The top portion of Figure 5 shows one critical change—the mean trial on which the model first formed a stable WM peak for the familiar item. A stable WM peak was defined as sustaining suprathreshold activity across the inter-stimulus interval (4 s; see Perone and Spencer, 2013a). Over development, the term (**A**) and preterm (**B**) infant models form WM peaks more quickly, with the preterm model lagging the term model. This index of the model's performance is important because maintaining the familiar item in WM produces strong inhibition in PF at sites involved in encoding the item. This, in turn, leads to less looking to the familiar item and more looking to the novel item. That is, quick WM formation allows the model to actively recognize what is known and seek novelty. In addition, quick WM formation leads to more frequent gaze switching and shorter look durations over development, allowing the model to more effectively explore items in the task space.

What drives these changes in WM in the model? These developmental differences emerge from the accumulation of distributed memory traces in HL_PF_ and HL_WM_ over time. Figures 5C,D shows the state of HL_PF_ and HL_WM_ for the 5- (blue lines), 7- (red lines), and 12-month-old (black lines) models. Over development, activation across the dimension grew in strength for the term (**C**) and preterm (**D**) models. In other words, the model became increasingly familiar with the entire dimension. This, in turn, led PF to encode items more quickly and WM to maintain those items more robustly.

These simulations shed new light on the origins of intrinsic motivation. Specifically, the simulations allow us to ask where the motivation to seek novelty comes from. Novelty seeking has some distinct advantages over familiarity seeking for infants. For example, novelty seeking enables infants to compare known with unknown items, efficiently explore complex environments, and, more generally, opens the door to discovery. Critically, infants do not know this ahead of time. Our simulations indicate that the motivation to seek novelty emerges from the accumulation of visual experience within a complex, dynamical exploratory system. A key property of the DNF model is that real-time, autonomous exploratory behavior creates a history that influences the behavior of the system at future points in time. The accumulation of this history over time led to the emergence of a new ability—quickly forming working memories of “known” items. This cognitive ability enables an increasing bias to seek novelty to gradually emerge without an explicit motivational force. We discuss this topic further in the General Discussion.

These simulations also shed new light on the population differences in the familiarity-to-novelty shift. In particular, the Hebbian traces accumulated for the term and preterm model were quite similar (compare Figures 5D,E) and were not sufficient to overcome the weaker neural interactions in the preterm infant model. This indicates that population differences in visual exploration and WM formation are largely attributable to the initial conditions of the system, while developmental changes emerge from the accumulation of out-of-the-lab experiences. Below, we probe whether altering the experience of the preterm infant model during development influences its novelty seeking behavior in the processing speed task.

#### Intervention

The simulations results described above show that novelty seeking emerges as experience accumulates via a Hebbian learning process. However, the initial conditions of the model played a major role in development: the accumulation of experience did not enable the preterm model to overcome the initially weaker neural interactions. How strong is this constraint on development? Are there ways that we might enhance the model's experience and, in turn, foster the development of novelty seeking biases?

There is a large literature showing that how other agents (e.g., parents) interact with infants while exploring objects influences how they distribute their looks (Landry and Chapieskie, 1988; Perrinello and Ruff, 1988). This is especially salient in interventions with preterm infants. For example, Landry et al. (2006, 2008) have shown that preterm infants benefit in the social, cognitive, and linguistic domains when parents are trained to act responsively to their infants while exploring objects as part of an intervention. This involves “following in” on the objects infants explore and helping infants maintain attention (e.g., by manipulating the object of infants' focus) rather than shifting attention to other objects (e.g., manipulating an object elsewhere).

Can we manipulate the nature of the preterm model's experience and transform it into a term-looking model in a similar way? For example, can we bias the model to continue looking at an object and, in turn, enhance encoding, WM, and long-term memory formation? Could this enhance traces in HL_PF_ and HL_WM_ enough to overcome the weaker neural interactions of the preterm model? This would help us assess the relative impact of the model's initial parameter setting versus the accumulation of out-of-lab experiences.

To test this possibility, we re-simulated the development of the preterm model. After the fifth episode, we implemented an intervention. We wanted to probe how an intervention might unfold in the real world where infants do some developing during the first few months of life, undergo assessment, and are assigned to an intervention thereafter. In our intervention, the model was biased to sustain looking at whatever item it happened to be fixating. If the model was fixating the left location, for example, the input from the object in at that location in space was increased. This, in turn, provided a slight boost of excitation to the fixation system, helping to maintain fixation. In the DNF model, this is equivalent to another agent manipulating an object in space (see Figure 1).

Figure 4 shows the simulation results. The intervention had the most dramatic impact on the number of trials to criterion (**E**) and looking to the familiar item (**F**). In particular, by 12 months the intervention model met criterion at a rate comparable to the term model at the same age. Similarly, by 12 months the intervention accumulated less time looking to the familiar item much like the term model at the same age. A substantive amount of intervention experience was required for the intervention to exert its effects on the model's performance in the processing speed task. Ultimately, however, the intervention created a preterm infant model with a robust novelty-seeking bias comparable to term infants.

What are the sources of these behavioral changes? Figure 5C shows the trial on which the intervention model formed a stable WM peak. At 5 (blue bars) and 7 (red bars) months, the intervention model formed a WM peak at rates comparable to the preterm infant model (**B**). By 12 months (black bars), however, the intervention model formed a WM peak at rates that exceeded the term model (**A**). This improved capacity of the intervention model to quickly encode items and maintain items in WM arises from the strength of activity accumulated in the HL. As can be seen **5D**, the strength of HL_PF_ and HL_WM_ by 12 months (black lines) is much stronger than at the same time for the term (**C**) and preterm (**D**) infant models. This stronger accumulation of activity in the HL enabled the intervention model to overcome the weaker neural interactions of the preterm infant model.

#### Spatial precision hypothesis

In our previous work, we implemented the SPH by hand, showing that stronger neural interactions lead the DNF model to form working memories more quickly (Perone and Spencer, 2013a). This effective increase in processing speed also led to stronger biases for novelty, shorter looks, and higher rates of gaze shifting. Here, we observed these very same patterns of change over development. But does the accumulation of experience via Hebbian learning yield the same changes in neural interactions produced by SPH?

Implementing the SPH via hand-tuning neural interactions leads to stronger, narrower WM peaks with deep lateral inhibition (see Schutte and Spencer, 2009). We tested whether the accumulation of experience in the HL reproduced this activation profile by initializing the DNF model with the state of the HL after 5, 10, 15, 20, and 25 episodes of exploration. The model was presented with a single stimulus for 2000 time steps. When the stimulus was removed, we sampled the state of WM every time step for 1000 time steps. We then averaged the state of WM across all samples to obtain a representative WM profile. Noise was turned off so that we could obtain a clean estimate of how the HL impact WM peaks (see Schutte and Spencer, 2009).

**Figure 6:**
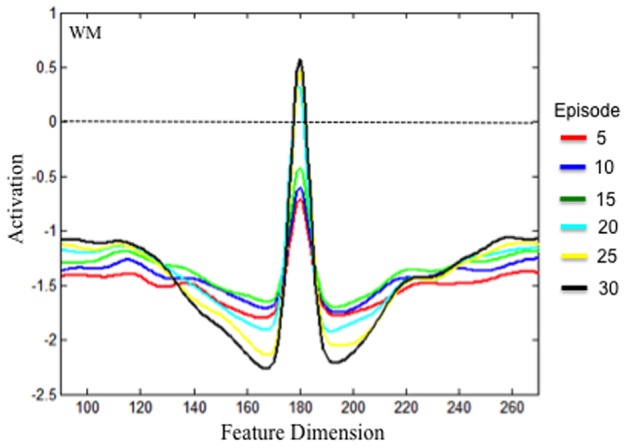
**Test results of whether experience accumulated across a dimension can lead to the SPH at the level of neural interaction.** The model was initialized with the experience accumulated in the Hebbian layers after every 5 episodes of exploration, which is shown by the different colored lines. The figure shows the state of WM during the inter-stimulus interval following stimulus presentation (see text). With experience, the WM field was able to form a stable peak. This peak had a strong excitatory component and deep inhibitory component much like implementing the SPH via hand-tuning the strength of excitatory and inhibitory connections.

The results are shown in Figure 6. Over development, the strength of the activation peak increased. After 5 (red), 10 (blue), and 15 (green) episodes of exploration, the peak was too weak to maintain a stable WM peak under the task conditions. After 20 episodes of exploration (cyan), the accumulated memory traces in HL_WM_ enabled WM to maintain a peak at suprathreshold (>0) levels. The model effectively acquired a new cognitive ability. In addition, the excitatory component of the peak grew in strength and became somewhat narrower over development. The inhibitory component grew broader and deeper as well. It is notable that these neurodevelopmental changes in excitation *and* inhibition were all driven by the accumulation of excitatory memory traces. As the strength of HL_WM_ increased, excitation in WM became stronger which passed stronger activation into the layer of inhibitory interneurons. This, in turn, projected stronger lateral inhibition back to WM. Thus, the present simulations demonstrate that the SPH can emerge over development via a variant of Hebbian learning as the model accumulates “out-of-the lab” experiences.

Simulation Experiment 1 revealed three key insights. First, the accumulation of visual experience along a dimension leads to quicker WM formation for stimuli on a familiar dimension. This quick recognition, in turn, promotes novelty-seeking. Second, the impact of visual experience on cognition is influenced by the initial state of the neurocognitive system. The neurocognitive deficits of the preterm infant model were expressed over development, leading to slower WM formation along a familiar dimension across the first year. Increasing the intensity of the experience the preterm infant model acquired with a dimension, however, enhanced WM formation by strengthening the familiarity with that dimension. Lastly, the accumulation of visual experience led to stronger neural interactions within the neural populations involved in encoding and WM formation. This strengthening was created by the accumulation of Hebbian learning but resembled the SPH at the neurocognitive (faster WM formation) and behavioral (less looking to familiar items) levels. These results indicate that processing speed and, consequently, the transition to novelty seeking over development emerges from experience.

## Simulation experiment 2

Simulation Experiment 1 showed that the familiarity-to-novelty shift emerges over development as experience accumulates via a Hebbian learning process. It also showed that the motivation to seek novelty comes for free from the dynamics of a historical cognitive and behavioral system. But these simulations were at the level of the group. Recall we simulated the development of 5 individuals and initialized the model in the processing speed task with the average state of those individual HL. The motivation to push boundaries in development, however, happens at the level of the individual. Each individual must forge a unique path and strive beyond what is currently possible.

In the infant cognition literature, individual differences in visual exploration have long been attributed to differences across infants in the neurodevelopmental mechanisms that underlie basic perceptual and cognitive processes (Colombo and Mitchell, 1990; Rose et al., 2007). This position stems from two observations. First, numerous studies have shown that individual differences in looking are stable during the first year of life (Colombo et al., 1987; Rose et al., 2001). Second, individual differences in looking are predictive of cognitive developmental outcomes in toddlerhood (2009) and adolescence (2012).

This view of individual differences is generally consistent with the group-level differences from Simulation Experiment 1. There, differences across simulations reflected, in part, different initial conditions in parameter values. Applied at the level of individuals, we might create an entire ensemble of individual models, with some models starting off with slightly stronger excitatory and inhibitory interactions than others (see, e.g., Perone and Spencer, 2013a). But individual differences might also reflect the differential accumulation of experience over development. For instance, Perone and Spencer (2013a,b) showed that experience on the task time scale creates variation in looking that mimics aspects of developmental changes, even when models start with the same initial conditions. Might the accumulation of experience over development lead to stable individual differences even when models—or infants—start out in the same neurodevelopmental state? We probe this possibility below.

### Method

We simulated the development of 10 individuals term, preterm, and intervention models using the same method described above with one exception. In the simulations above, we averaged the HL across simulations and situated the model in the processing speed task after 5, 10, and 30 episodes. Here, we initialized the model with HL_PF_ and HL_WM_ from each of the 10 individual simulations. As above, each model was run in the processing speed task 100 times to assess the full range of performance for each individual.

### Results and discussion

**Figure 7:**
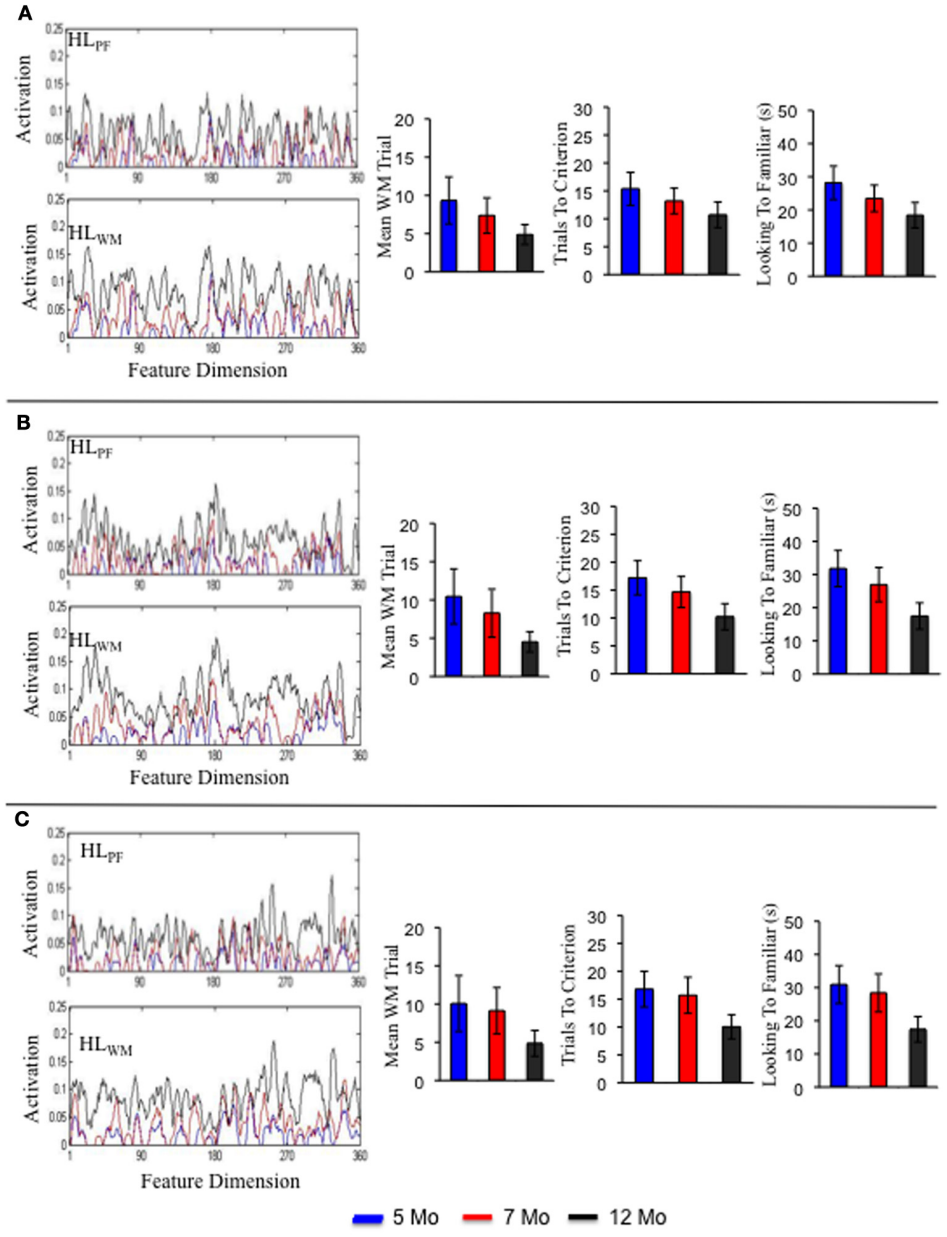
**Shows the Hebbian layers and performance in the processing speed task for three individual simulations of the term model.** Each panel shows an individual simulation at 5 months (blue), 7 months (red), and 12 months (black).

Figure 7 shows a sample of three individual term infant simulations. The left portion shows the activation traces in HL_PF_ and HL_WM_ for each simulation. Notice that each simulation varies in the strength, distribution and location of peaks and valleys along the feature dimension. Also notice that these peaks and valleys are much more pronounced at the individual level than at the group level (compare **7A** to **5C**). This highlights individual differences in what the model happened to form robust memories for during its development. The right side of the figure shows three measures from the processing speed task: trial of stable WM peak formation, trials to criterion, and looking to the familiar. As can be seen, each individual follows a distinct, yet similar, developmental trajectory. For example, the individual in **7A** showed a shallow, steady decrease in the trials to meet criterion over development. The individual in **7B** showed a steep decline. And the individual in **7C** showed little decline from 5 to 7 months but a sharp decline from 7 to 12 months.

**Figure 8:**
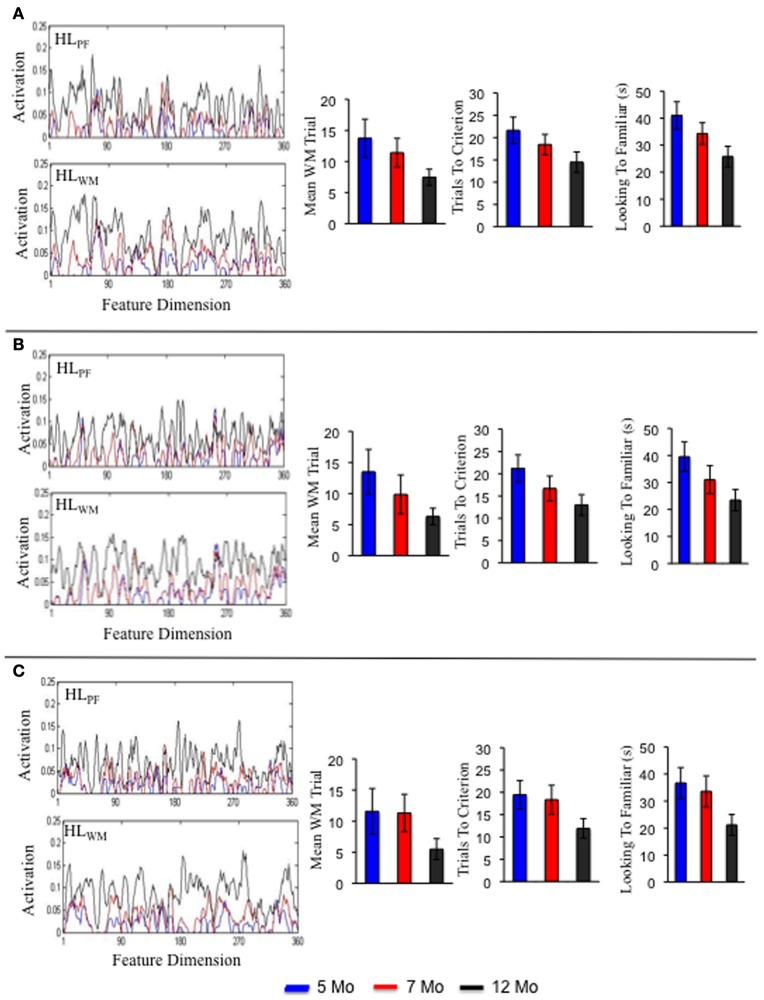
**Shows the Hebbian layers and performance in the processing speed task for three individual simulations of the preterm model.** Each panel shows an individual simulation at 5 months (blue), 7 months (red), and 12 months (black).

**Figure 9:**
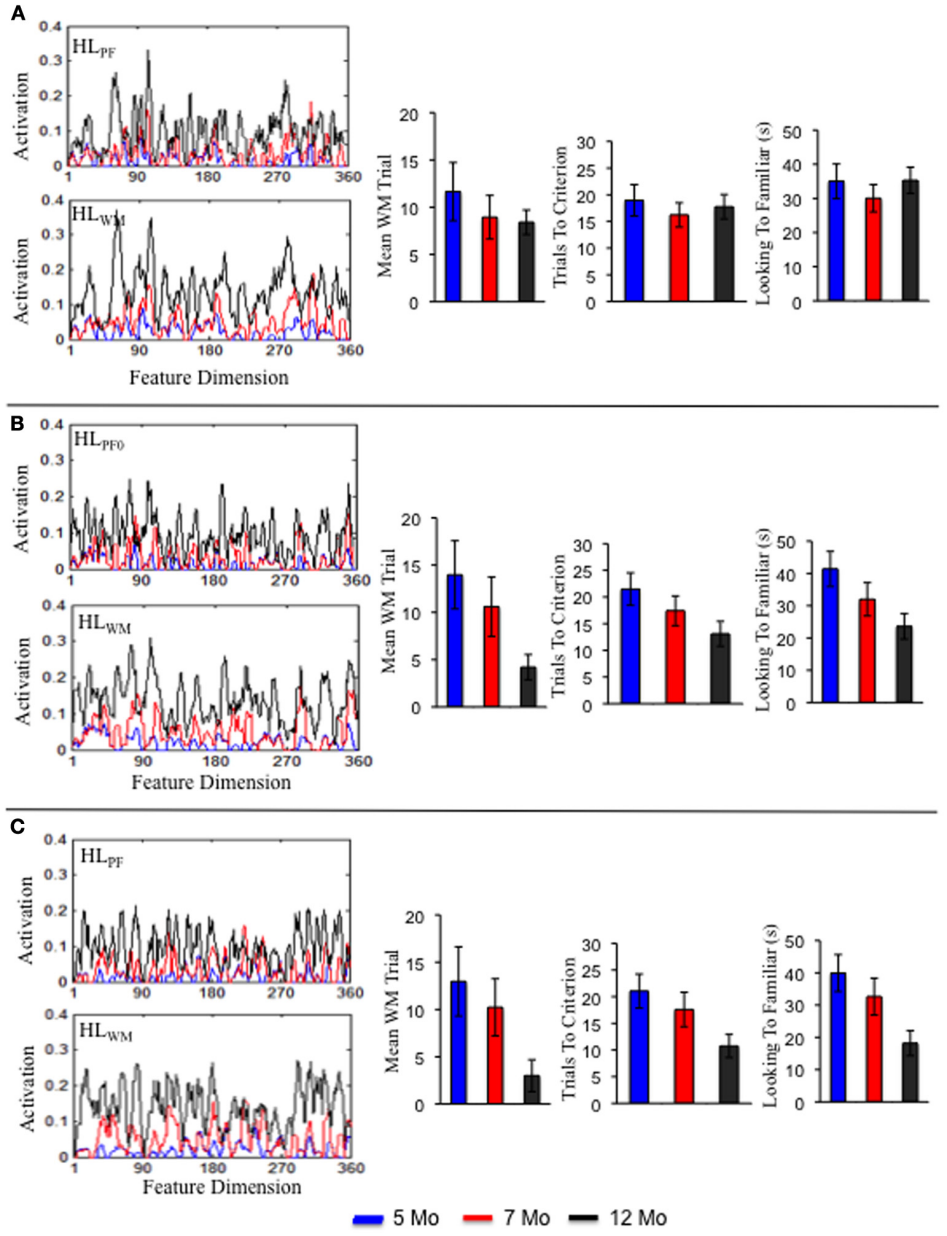
**Shows the Hebbian layers and performance in the processing speed task for three individual simulations of the intervention model.** Each panel shows an individual simulation at 5 months (blue), 7 months (red), and 12 months (black).

This holds true for the preterm infant model as well. Three individual simulations of this model are shown in Figures 8A–C. Consistent with the group level simulations, the structure of the developmental trajectories for the individual term and preterm infant models were influenced by the initial conditions. That is, individual preterm infants exhibited a similar, yet delayed, developmental trajectory relative to the individual term infant models. The pattern is somewhat different for the intervention model. Three individual simulations of this model are shown in Figures 9A–C. For the intervention simulations, some individuals showed a dramatic decline in trials to criterion by 12 months of age, much like the group level analyses (see **9C**). Others, by contrast, showed an increase in the number of trials to criterion (see **9A**).

**Figure 10:**
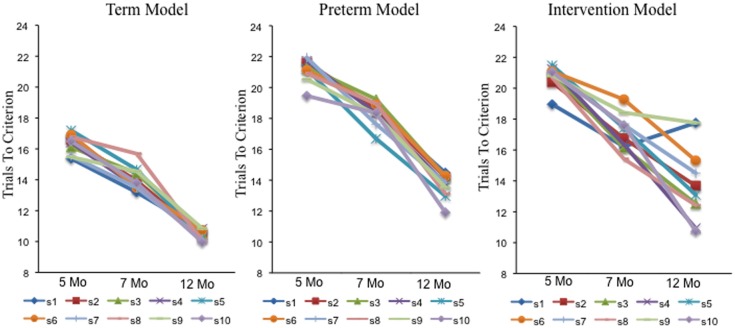
**Shows trials to criterion for 10 individual simulations at 5, 7, and 12 months for the term (left), preterm (middle), and intervention (right), models**.

Figures 7–9 show that each individual had a unique developmental trajectory. But did the accumulation of experience in the model create a stable pattern of familiarity and novelty seeking biases over development? In other words, were familiarity-seeking individuals early in development also familiarity-seeking individual later in development? Figure 10 shows the trials to criterion for the 10 individual term, preterm, and intervention simulations. Inspection of the plots reveals some stability over development in each group, even though individual runs of the model in each group had exactly the same initial conditions. For the term infant model, S8 (salmon) and S5 (turquoise) are relatively slow processors at 5 and 7 months. S1 (blue) and S7 (light blue) are fast processors at 5 and 7 months. And S3 is neither fast nor slow at 5 and 7 months. The preterm infant model is considerably more variable. The weaker neural interactions of the preterm model make it more susceptible to stochastic influences. Nevertheless, S3 (green) and S6 (orange) are faster than S9 (light green) and S10 (purple) at all three ages. The intervention model was even more variable than the preterm infant model, yet it also showed signs of stability. For example, S10 (light purple) was faster than S6 (orange) at all three ages.

The striking variability in the individual intervention simulations indicates that the intervention did not impact every individual in the same way. For example, S4 (dark purple) and S10 (light purple) were both quick novelty-seekers by 12 months. By contrast, S1 (blue) quickly met the novelty-seeking criterion at 5 months but exhibited in an increase the trials to criterion at 12 months. Figure 8A shows the accumulation of activation in the HL for this model. As can be seen, S1 acquired some tall, broad memory traces (see near site 80) between 7 (red line) and 12 (black line) months in both HL. This pattern of activity can lead to the model to dwell because the traces in PF are so strong. Consequently, the model spent more time looking to the familiar item and exhibited longer look durations at 12 months (black bars) than at 7 months (red bars) even though it actually formed a WM peak more quickly at 12 months than at 7 months. The accumulation of activity in the HL for S5 and S10 are shown in Figures 8B,C, respectively. These simulations acquired a more evenly distributed pattern of activity, especially in HL_PF_. This, in turn, led these simulations to exhibit a relatively consistent shift from familiarity to novelty seeking over development that aligned well with their developing capacity to form working memories. These simulation results raise the exciting possibility that we can map individual models to individual infants and capture the impact of real-world interventions. We return to this issue below.

The results from individual simulations suggest that individual experiences can give rise to stable individual differences over development. To quantify this across the full set of simulations, we used hierarchical regression. Table 1 shows the regression analysis. The table presents the predictor variables entered on each step and a number of summary statistics. On the left are the proportion of variance accounted for by the predictors (*R*^2^), change in proportion of variance accounted for across steps (change in *R*^2^), change in the *F* statistic across steps, and the probability associated with the *F* statistic. On the right are the unstandardized beta weights (ß) and standardized beta weights (beta). The weight is the unique contribution of each predictor. The sign indicates the direction of the relationship between the predictor and dependent measure. The size of the weight indicates the slope. Steeper slopes indicate that the dependent measure changes more for each unit change in the predictor. The *p* value shows the statistical significance of each predictor.

**Table 1:** **Predicting trials to criterion at 12 months**.

**12 months trials to criterion**								
**Step**	**Predictors**	***R*^2^**	***R*^2^ change**	***F* change**	***p***	**β**	**Beta**	***p***
1	Model group	0.39	0.39	17.88	<0.01	1.67	0.62	<0.01
2	5 months criterion	0.57	0.19	5.64	0.01	−0.24	−0.26	0.46
	7 months criterion					0.73	0.68	0.02

In the first step, we controlled for group by entering group (term = 1, preterm = 2, and intervention = 3) as a predictor and trials to criterion at 12 months of age as the dependent measure. Group accounted for a significant proportion of variance in trials to criterion at 12 months of age, *R*^2^ = 0.39. In the second step, we entered trials to criterion at 5 and 7 months. Trials to criterion early in development did account for a significant proportion of variance at in trials to criterion later, *R*^2^ Change = 0.19. Evaluating the beta weights indicts that trials to criterion at 7 months of age was the strongest predictor. The positive slope of the beta weight indicates that more trials to criterion at 7 months of age was associated with more trials to criterion at 12 months of age. In the past, we found that experience in the DNF model on the task time scale leads to patterns of covariation between looking and novelty preferences like real infants. These results provide compelling evidence that experience creates stability on the developmental time scale in familiarity and novelty seeking behavior at the level of the individual.

## General discussion

Children make astonishing transformations during just a short period of time, raising the question of why they continually strive forward in development. Examining the sources of intrinsic motivation early in development might offer a particularly compelling case that provides insights into the very origins of motivational states. Here, we examined a key transition in exploratory biases in the first year of life as infants move from familiarity-seeking to novelty-seeking. This familiarity-to-novelty shift emerges gradually over the first year, differs across infant populations, and is stable within individuals over time (see Hunter and Ames, 1988; Rose et al., 2001, 2002, 2007). Novelty seeking has some distinct advantages. For example, it allows infants to compare and contrast known items in memory with new items in the environment (Oakes et al., 2008). This might help them form categories and inspect multiple items before deciding to approach them for further exploration. But what motivates the infant to switch exploratory styles?

To address this question in the present report, we used a DNF model of infant visual exploration that has accounted for the familiarity-to-novelty shift in previous work (Perone and Spencer, 2013a,b). Previous findings showed that when we implemented the SPH “by hand” over development, the DNF model could capture the qualitative and quantitative aspects of this shift. This included examples of infants' robust familiarity preferences during the first two months of life (Wetherford and Cohen, 1973; see also Fantz, 1974), as well as the more gradual increase in novelty seeking over the course of the first year. Here, we asked if the DNF model could transform itself from a familiarity to novelty seeking model through nothing more than “out-of-lab” experience. Our strategy was to let the DNF model accumulate experience in HL via autonomously exploring a virtual world consisting of objects distributed over a continuous feature dimension. We then asked whether the model exhibited the familiarity-to-novelty shift in the processing speed task.

Our results show that the model can autonomously transform itself from a familiarity to novelty seeking model over development. As the model explored its virtual world, it accumulated traces in the HL. Over time, this experience helped the model quickly encode items and form stable WM peaks. This, in turn, enabled the model to actively represent known items and explore novel ones. Our results also showed that the initial conditions of the model created differences in the familiarity-to-novelty shift like those observed between term and preterm infants (Rose et al., 2002; see also Rose et al., 2001). Specifically, when we set the initial conditions of the preterm model to have weak neural interactions, the model shifted toward novelty more slowly over development, much like preterm infants do. Interestingly, we found that the experience the preterm infant model accumulated in the HL was comparable to the term infant model. This indicated that experience can create developmental change in the familiarity-to-novelty shift but the initial conditions play a major role in population differences.

Critically, these constraints are soft constraints: when we performed an intervention where we biased the model's pattern of looking, the developmental trajectory shifted in individual simulations. In particular, the intervention helped the models dwell on objects longer, creating stronger memory traces in the HL. In some models, this had advantageous effects: these models encoded items more quickly into WM and exhibited novelty-seeking behaviors late in the first year that mimicked the pattern of term infants. In other models, however, the Hebbian traces in the perceptual field became too strong and the models showed a developmental regression with a bias toward familiarity.

The large variability in the outcomes of the intervention models is consistent with recent intervention studies that have trained caregivers to maintain their infants' attentional focus on objects. These interventions have facilitated positive developmental outcomes for children in areas of language, coordinated joint attention, and increased frequency with which caregivers maintain attentional focus (Landry et al., 2008). Nevertheless, the impact of such intervention studies has been diluted by individual differences in infants and caregivers. For example, preterm infants who experienced severe neonatal complications do not benefit from caregiver responsiveness to the same degree as infants who experienced relatively less severe neonatal complications (Landry et al., 2006). To optimize intervention, then, we need to tailor intervention to individuals.

We suggest that the DNF model might be a useful tool in these efforts. For instance, our simulation results suggest that we could initialize models to capture the performance of very young preterm infants in standard laboratory tasks. Critically, we could initialize models to capture the performance of individuals, not simply groups. We could then simulate different long-term interventions with these models and observe the predicted outcomes. This could help researchers design individualized intervention regimens. Importantly, the models not only predict long-term outcomes, but also short-term benchmarks in performance. For instance, we could assess the models and infants at 3 months intervals in standard laboratory tasks to determine whether infants' looking and learning abilities match what is predicted by each infant's model. This provides multiple benchmarks to determine whether the intervention is on track.

Although the work presented here suggests that the DNF model could be a useful intervention tool, achieving this vision will require multiple layers of innovation. Most critically, the intervention we implemented was overly simplistic and ignores a fundamental factor in development—the role of other agents in infants' cognitive development. Infants develop in a rich social context that involves other agents such as parents and siblings. As described above, how other agents interact with infants while exploring objects can have a profound impact on how infants distribute their looks in time and in space as well as social interactions between infants and their caregivers (Perrinello and Ruff, 1988; see also Landry and Chapieskie, 1988). We are currently probing how a dyadic system that consists of parent and infant models sharing the same environment might explain the role of individual differences in parents and infants on the outcome of interventions as well as the emergence of social interactions in exploratory settings. This advancement will open the door to probe optimal intervention conditions for each parent-infant dyad. This may have far reaching practical implications.

Using the DNF model as an intervention tool in future work will also require tackling several challenges we simplified in the present simulation experiments. Conceptually, our model developed over the course of months. In practice, however, we simulated the model for much less time. This reflected the goals of this paper—to examine whether it was possible to have an autonomous model develop its own transition is visual exploratory biases. But using the model in more practical applications such as designing interventions will require that we more closely approximate the real-world experience of individual infants. We also encountered several practical challenges in the simulations that will be even more dramatic in more realistic simulation efforts. For instance, sometimes our models showed overly robust WM peaks that would endure for long periods of time. This would create a strong Hebbian trace that could dominate the looking and learning dynamics. We prevented this, in part, by carving the simulations up into episodes and re-initializing the layers every 10,000 time steps. In a more realistic model, we suspect this could be handled by adding more noise sources. For instance, data with infants suggests that their attentional abilities wax and wane over time (Oakes and Ross-Sheehy, 2004). We could implement this type of attentional inertia by adding a noisy resting level to the WM and PF layers that would gradually raise and lower slowly over time. The troughs in this type of attention would de-stabilize even robust WM peaks. This suggests that noise could serve an adaptive function in early development, facilitating exploration and ensuring that the system does not get stuck focusing too much on one thing.

This brings us back to the central issue we started with: what motivates infants to move from an initial bias toward familiarity to a robust bias toward novelty? In one sense, our simulations suggest that there is no motivational source that propels the system forward in development. The DNF model propels itself forward because it is a complex, exploratory, dynamical system that accumulates its own history over time. Each time the DNF model formed a WM peak, this neural event left a trace in HL_WM_. The accumulation of this history over time raised the overall excitability of the WM field, leading to more robust WM peaks and the active maintenance of familiar items. This qualitatively new cognitive ability enabled the model to actively recognize what is known and explore new items in the environment. Thus, our autonomously developing model shows how changes in infants' visual exploratory skill measured in laboratory tasks can emerge from the accumulation of experience outside of the lab. There is no special motivating force that propels the model forward through development; rather, exploration and skill development come “for free” given the complex, self-organizing neural dynamics of the visual exploratory system. This is nicely illustrated by the full range of simulations we reported. Not all of our simulations developed a novelty bias—at least one of the intervention simulations showed a developmental regression, returning to familiarity-seeking behavior.

We contend that exploration is a fundamental, emergent property of complex dynamical systems—such systems can't help but explore (Thelen and Smith, 1994). In particular, given the high-dimensional nature of coupled behavioral and neural systems, such systems are inherently variable as they exchange energy with the surrounds and pass activation back-and-forth among different components of the system (Kelso, 1995). Such systems are also self-organizing, routinely settling in temporarily stable organizational states. Exploration emerges from the inherent tension between stability and variability. And in high-dimensional systems, this tension inevitably leads to new possible patterns of organization. Critically, complex dynamical systems are also historical, carrying this history forward through time. This sets the stage for new organizational patterns to be continually revisited and re-evaluated. Selection of adaptive states can then occur (Edelman, 1987).

There is another sense, however, in which our simulations suggest a motivational source is at work as infants transition from familiarity- to novelty-seeking. Oudeyer and Kaplan (2007) proposed two characterizations of intrinsic motivation. The first was a force that propels development forward, the notion of intrinsic motivation that is common in developmental psychology. As described above, this source was seemingly absent from the DNF model as it transitioned from familiarity- to novelty-seeking. The second characterization was in terms of the neurocognitive mechanisms that drive action. Conceptually, the idea is that subjective experiences of interestingness, ambiguity, and surprise move one to act. These subjective experiences might be driven by several neurocognitive mechanisms. Interestingness, for instance, can be driven by the degree to which an expected and experienced outcome differs. This sense of intrinsic motivation is present in the DNF model. Specifically, the pattern of connectivity among the layers of excitatory and inhibitory neurons in the model implements a neurocognitive mechanism that can identify “known” from “unknown”—“expected” from “unexpected”—and then drive exploratory behavior.

If intrinsic motivation is inherent in the architecture of the model, a central question is where this architecture comes from. In our simulations, the architecture is assumed to be present early in development. Data are consistent with this conjecture. For instance, newborns exhibit evidence of recognizing stimuli experienced prenatally (DeCasper and Spence, 1986). But such data merely shifts the question of origins earlier. In our view, the neural architecture we proposed is likely a result of early prenatal developmental processes that are heavily dependent on patterned neural activity. For instance, recent work suggests that the type of neural architecture used here—DNFs—can emerge from a self-organizing process (Alecu et al., 2011; Detorakis and Rougier, 2012). Thus, the type of connectivity we assumed does not have to be “hard wired” in any sense—it can emerge during the course of early brain development. This also suggests that the neural system we proposed might be ubiquitous across species, consistent with evidence showing novelty-seeking behaviors in rabbits (Smith and Litvaitis, 2000), birds (Blough, 1984), and squirrels (Duncan and Jenkins, 1998).

In this context, it is important to note that novelty-seeking might not be the only outcome of autonomous visual exploration. In some studies, infants, and even adults, seek familiarity for items they do in fact have a robust memory for (Dodd et al., 2009). Seeking familiarity is clearly valuable in achieving practical goals—we often search for our coffee mug, keys, and so on. We are currently exploring how the DNF model might organize itself as a familiarity-seeking model in some contexts and novelty-seeking model in others.

In summary, a robust developmental trend in infants' visual exploration is that infants transition away from familiarity and toward novelty. This trend has largely been described as a by-product of faster processing speed; as processing speed increases, new items become familiar more quickly to infants and they are free to explore novelty. Our simulations indicate that novelty seeking and processing speed mutually support the development of each other. As infants explore more items along a dimension, they become increasingly familiar with that dimension. This, in turn, enables them to quickly form memories for items on that dimension and continue to explore novelty. We gained this insight by using a DNF model of infant visual exploration to ask what motivates an infant to switch their exploratory style from familiarity- to novelty-seeking. The DNF model propelled itself forward simply by autonomously accumulating a learning history as it explored a virtual visual world with a reasonable degree of stimulus variation. In this sense, no motivational force was required for the model to shift its exploratory style. In another sense, however, the pattern of neuronal connectivity in the model clearly sets the stage for this shift to happen. Most critically, our simulations suggest that the accumulation of real-time exploratory behavior is powerful enough to create developmental change.

### Conflict of interest statement

The authors declare that the research was conducted in the absence of any commercial or financial relationships that could be construed as a potential conflict of interest.

## Appendix

The notation used in the equations is presented in Table A1.

**Table A1:** **Notation**.

**Letter**	**Meaning**
*a*	Amplitude/strength parameter
*x, y*	Dimension (*x* = color, *y* = shape)
*l*_*i*_	Looking nodes (*i* = index of the node)
*u*	Activation variable for PF
*v*	Activation variable for Inhib
*w*	Activation variable for WM
*m*	Activation variable for memory/Hebbian layer
*s*	Stimulus input (Gaussian for fields)
*c*	Connection weight function
*g*	Gating function
*t*	Time
τ	Time scale parameter
*h*	Resting level (static or dynamic)
*n*	Number of nodes
*r*	Random contribution
ξ	Noise parameter
*e*	Excitatory
*i*	Inhibitory

### Model equations

Each neuronal layer is specified by a differential equation numerically integrated using the Euler method.

#### Perceptual field (PF)

PF consists of reciprocally coupled excitatory, PF(*u*), and inhibitory, Inhib(*v*), layers for dimension *x*. The excitatory layer of PF is given by the following equation:

$$\begin{array}{l} \tau_{e}\dot{u}(x,t)=-u(x,t)+h_{u}\\ \;+a_{ul}\sum_{\dot{l}=1}^{n}g(l_{i})\sum_{\dot{l}=1}^{n}s_{i}(x,t)g(l_{i})\\ \;+\int c_{uu}(x-x^{\prime })g(u(x^{\prime },t))dx^{\prime }\\ \;-\int c_{uv}(x-x^{\prime })g(v(x^{\prime },t))dx^{\prime }\\ \;-a_{\text{uv}\_\text{global}}\int g(v(x^{\prime },t))dx^{\prime }\\ \;+\int c_{um}(x-x^{\prime })m(x^{\prime },t)dx^{\prime }\\ \;+\int c_{r}(x-x^{\prime })\xi (x^{\prime },t)dx^{\prime } \end{array}$$

where $\dot{u}(x,t)$ is the rate of change of activation in the excitatory layer of PF across the continuous behavioral dimension, *x*, as a function of time, *t*. τ_*e*_ is the time constant along which excitatory activation evolves. Activation within PF is influenced by its current state, *u*(*x, t*), and its negative neuronal resting level, *h*_*u*_. PF receives a global boost from the fixation system, $a_{ul}\sum_{\dot{l}=1}^{n}g(l_{i})$, which is dictated by the gating function, *g*(*l*_*i*_), and weighted by the amplitude or “strength” parameter, *a*_*ul*_. This means that when a task-relevant location is fixated, PF receives a boost of activation. PF also receives stimulus input at the suprathreshold fixated location, $\sum_{\dot{l}=1}^{n}s_{i}(x,t)g(l_{i})$, where *s*_*i*_(*x*,*t*) is a Gaussian input (see below) distributed across the behavioral dimension, *x*. Note that for these inputs *n* = 2 because only looking nodes associated with the left and right locations are associated with task-relevant stimuli in the task space (see “Fixation System” below).

The gating function is given by the following equation which takes a sigmoidal shape over the activation variable, *u*:

$$g(u)=\left[\frac{1}{1+\exp\left[-\beta (u(t)-u_{0})\right]}\right],$$

where β is the slope of the sigmoid function and *u*_0_ is the threshold (0).

The stimulus input takes the form of a Gaussian distributed over the behavioral dimension, *x*:

$$s(x,t)=a\exp\left[-\frac{{\left(x-\mu \right)}^{2}}{2\sigma^{2}}\right]\chi (t)$$

with stimulus position centered at μ, strength *a* (set to 17), and width σ (set to 3). The gating function, χ(*t*), is set to 1 when the stimulus is present and 0 otherwise.

PF dynamics are also influenced by local excitatory within-layer interactions, ∫ *c*_*uu*_(*x* − *x*′)*g*(*u*(*x′, t*))*dx′*. These interactions are specified by the convolution of a Gaussian profile, *c*_*uu*_(*x* − *x′*), which determines the neighborhood across which excitatory interactions propagate and a non-linear gating function, *g*(*u*(*x*′, *t*))*dx*′, dictating that only neurons with above threshold activation (>0) participate in the interactions.

The Gaussian convolution was defined by:

$$c(x-x^{\prime })=a\exp\text{​}\left[-\frac{{\left(x-x^{\prime }\right)}^{2}}{2\sigma^{2}}\right]$$

where *a* sets the amplitude and σ sets the width (i.e., standard deviation) of the connection matrix function.

PF dynamics are also influenced by two inhibitory components. The first is a local inhibitory component, ∫ *c*_*uv*_(*x* − *x′*)*g*(*v*(*x′, t*))*dx′*. Inhibitory interactions are projected across a neural neighborhood specified by a Gaussian, *c*_*uv*_(*x* − *x′*), and only above-threshold activity in the inhibitory layer contribute to interactions. The second is a global inhibitory component, *a*_uv_global_ ∫ *g*(*v*(*x′, t*))*dx′*, where the sum of suprathreshold activity within the inhibitory layer across the behavioral dimension, *x*, at time, *t,* is weighted by *a*_uv_global_.

The last contribution to PF dynamics is spatially correlated noise, which is presented to PF by convolving a field of white noise with a Gaussian kernel, ∫ *c*_*r*_(*x* − *x′*)ξ(*x′, t*)*dx′*, with strength, *a*_*r*_, set to.025 and width, σ_*r*_, set to 1.

#### Inhibitory field (Inhib)

The excitatory layers PF(*u*) and WM(*w*) are reciprocally coupled to an inhibitory layer, Inhib(*v*). The equation for Inhib is:

$$\begin{array}{l} \tau_{i}\dot{v}(x,t)=-v(x,t)+h_{v}\\ \;+\int c_{vu}(x-x^{\prime })g(u(x^{\prime },t))dx^{\prime }\\ \;+\int c_{vw}(x-x^{\prime })g(w(x^{\prime },t))dx^{\prime }\\ \;+\int c_{r}(x-x^{\prime })\xi (x^{\prime },t)dx^{\prime } \end{array}$$

where $\dot{v}(x,t)$ specifies the rate of change of activation for each neuron along the behavioral dimension, *x*, as a function of time, *t*. τ_*i*_ is the time constant along which inhibitory activation evolves. Activation in Inhib is influenced by its current state, *v*(*x, t*), and its resting level, *h*_*v*_. Inhib receives excitatory inputs from PF, ∫ *c*_*vu*_(*x* − *x′*)*g*(*u*(*x′, t*))*dx′*, and WM, ∫ *c*_*vw*_(*x* − *x′*)*g*(*w*(*x′, t*))*dx′*. These inputs are projected across a neural neighborhood specified by a Gaussian projection, *c*(*x* − *x′*), to which only suprathreshold neurons in PF and WM contribute as dictated by the gating function, *g*. An independent source of spatially correlated noise is also added to the inhibitory layer, ∫ *c*_*r*_(*x* − *x′*)ξ(*x′, t*)*dx′*.

#### Working memory field (WM)

The WM(*w*) field is given by the following equation:

$$\begin{array}{l} \tau_{e}\dot{w}(x,t)=-w(x,t)+h_{w}\\ \;+a_{ws}\sum_{\dot{l}=1}^{n}s_{i}(x,t)g(l_{i})\\ \;+\int c_{wu}(x-x^{\prime })g(u(x^{\prime },t))dx^{\prime }\\ \;+\int c_{ww}(x-x^{\prime })g(w(x^{\prime },t))dx^{\prime }\\ \;+\int c_{wv}(x-x^{\prime })g(v(x^{\prime },t))dx^{\prime }\\ \;-a_{\text{wv}\_\text{global}}\int g(v(x^{\prime },t))dx^{\prime }\\ \;+\int c_{wm}(x-x^{\prime })m(x^{\prime },t)dx^{\prime }\\ \;+\int c_{r}(x-x^{\prime })\xi (x^{\prime },t)dx^{\prime } \end{array}$$

The equation for WM is identical to the equation for PF with two exceptions. First, the input from the fixation system differs: there is no global boost in activation from the fixation system into WM, and the stimulus input to WM, $a_{ws}\sum_{\dot{l}=1}^{n}s_{i}(x,t)g(l_{i})$, is weighted by a strength parameter, *a*_*ws*_, which was set to.05. Second, WM receives an excitatory input from PF, ∫ *c*_*wu*_(*x* − *x′*)*g*(*u*(*x′, t*))*dx′*.

#### Memory/hebbian layers (HL)

Activation in PF and WM is influenced by traces in an associated memory (m) or Hebbian layer (HL), which implement a form of Hebbian learning (see text). The equations for each HL are identical. The equation for the HL associated with PF is:

$${\dot{m}}_{u}(x,t)=\{\begin{array}{ll} (-m_{u}(x,t)+g(u(x,t))/\tau_{\text{m}\_\text{build}} & if\;u(x,t)>0\\ (-m_{u}(x,t))/\tau_{\text{m}\_\text{decay}} & \text{otherwise} \end{array}$$

where ${\dot{m}}_{u}(x,t)$ is the rate of change of activation for each site, *x*, in HL as a function of time, *t*. The constants τ_m_build_ and τ_m_decay_ set the time scale along which activation traces accrue and decay, respectively. Activation in HL only accrues when there is suprathreshold activation in PF. Otherwise, activation in HL decays.

#### Fixation system

The fixation system consists of four nodes that stochastically look at left and right locations (at which stimuli can appear) and center and away locations (at which no task-relevant stimuli appear). The nodes interact in a mutually inhibitory, winner-takes-all fashion. The equation for the fixation system is:

$$\begin{array}{l} \tau_{e}{\dot{l}}_{i}(t)=-l_{i}+h_{i}(t)+s_{i}(t)\\ \;+a_{ii}g(l_{i})\\ \;+a_{lu}g(l_{i})\int g(u(x^{\prime },t))dx^{\prime }\\ \;-a_{\text{l}\_\text{global}}\sum_{j\neq \dot{l}}g(l_{j}) \end{array}$$

where the activation variable, *l*, is set by the excitatory time scale, τ_*e*_. Activation of each looking node is influenced by its current state, *l*, and its dynamic negative resting level, *h*_*i*_(*t*) (described below). Activation of each looking node is also influenced by a stimulus input given by:

$$s_{i}(t)=a_{\text{i}\_\text{tonic}}(t)(a_{i}+\xi (t))+a_{\text{i}\_\text{transient}}(t)$$

The stimulus associated with each node is different (see “Fixation System Parameters” below) to reflect the different stimulus properties of the attention-getter at the central location, the stimuli at the left and right locations, and non-task-relevant input at all “away” locations. The left and right nodes are presented with a noisy input at each time step when a stimulus is present, *a*_i_tonic_(*t*)(*a*_*i*_ + ξ(*t*)), and a transient input to signify the appearance of a stimulus, *a*_i_transient_(*t*), present for the initial 75 time steps of each stimulus presentation. The away node is continuously presented with a noisy input to signify the “tonic” presence of stimuli in the task space. The center node is presented only with a transient input to reflect attention-getting stimuli briefly present at the onset of a trial (in our simulations, 50 time steps), effectively driving the fixation system to switch gaze from the away location to the center location.

The gating function, *g*, dictates the presence of a self-excitatory component to each looking node, *a*_*ii*_*g*(*l*_*i*_), and the passing of a negative, inhibitory input to all other nodes, $a_{\text{l}\_\text{global}}\sum_{j\neq \dot{l}}g(l_{j})$, with weight *a*_l_global_. The gating function also regulates the presence of input to the fixation system from the perceptual field across dimension *x, a*_*lu*_*g*(*l*_*i*_) ∫ *g*(*u*(*x′, t*))*dx′*, with weight *a*_*lu*_. Note that these inputs are set to 0 for the looking nodes associated with the center and away locations because there is no stimulus presented at those locations.

The resting level of each looking node is dynamic and is governed by the following equation:

$$\tau_{h}{\dot{h}}_{i}(t)=-h_{i}(t)+a_{\text{h}\_\text{rest}}+a_{\text{h}\_\text{low}}g(l_{i})$$

where τ_*h*_ sets the time scale along which the resting level of each node, *h*_*i*_, evolves. When the current level of activation of a looking node is above threshold [determined by the gating function, *g*(*l*_*i*_)] the resting level decreases toward a low attractor, the sum of*a*_h_rest_ and *a*_h_low_(which are both negative values). When the current level of activation of a looking node is below threshold, the resting level returns to baseline, *a*_h_rest_.

### Model parameters

Table A2 shows the parameters for the neurocognitive system and Table A3 shows the parameters for the fixation system used to simulate the looking behavior of term and preterm infants. To create the preterm infant model, we began with the term infant parameters and manipulated the parameters used to implement the SPH (see Schutte and Spencer, 2009; see also Perone et al., 2011; Perone and Spencer, 2013a,b). This involved uniformly decreasing the strength of within-layer excitatory connections in PF (*a*_*uu*_) and WM (*a*_*ww*_) and across layer inhibitory connections from inhib to PF (*a*_*uv*_) and to WM (*a*_*wv*_) by 20%. The SPH parameters are shown in bold. All other parameters were fixed for the term and preterm models. Note that for the intervention simulations (see text), 0.0625 was added to *a*_*i*_ when left or right node was supratheshold.

**Table A2:** **Neurocognitive system parameters**.

**PF(*u*)**			**WM(*w*)**			**Inhib(*v*)**			**Time scales (τ)**		**Memory layers (M)**	
	**Term**	**Preterm**		**Term**	**Preterm**		**Term**	**Preterm**				
*h*_*u*_	−10	–	*h*_*w*_	−5	–	*h*_*v*_	−10	–	τ _*e*_	80	*c*_*um*_	2.5
***a_uu_***	**0.75**	**0.6**	***a_ww_***	**2.0075**	**1.606**	***a_uv_***	**0.459**	**0.3672**	τ _*i*_	10	σ _*um*_	3
σ _*uu*_			σ _*ww*_	3	–	σ _*uv*_	10	–	τ_build_	20,000	*c*_*wm*_	1.5
			*a*_*wu*_	1.2	–	*a*_*vu*_	0.2	–	τ_decay_	400,000	σ _*wm*_	5
			σ _*wu*_	5	–	σ _*vu*_	5	–	τ _*h*_	80		
			*a*_*vw*_	4.5	–	***a_wv_***	**0.405**	**0.324**				
			σ _*v*__*w*_	5	–	σ _*wv*_	30	–				
						*a*_*vw*_	4.5	3.6				
						σ _*vw*_	5	–				
						*a*_*wv*_global_	0.01	–				
						*a*_*uv*_global_	0	–				

**Table A3:** **Fixation system parameters**.

**Location**				
	**Left**	**Right**	**Center**	**Away**
*a*_*l*_global_	1.8	–	–	–
*a*_*ii*_	2.00	–	–	–
*a*_*iu*_	0.25	–	–	–
*a*_*ui*_	1.00	–	–	–
*a*_*i*_transient_	3.00	3.00	15	0
*a*_*i*_tonic_	5.60	–	–	–
*a*_*i*_	0.70	–	–	–
*a*_*h*_rest_	−5.00	–	–	–
*a*_*h*_down_	−3.60	–	–	–
